# Possible Role of Minor H Antigens in the Persistence of Donor Chimerism after Stem Cell Transplantation; Relevance for Sustained Leukemia Remission

**DOI:** 10.1371/journal.pone.0119595

**Published:** 2015-03-16

**Authors:** Cornelis R. van der Torren, Yvette van Hensbergen, Susanne Luther, Zohara Aghai, Zuzana Stachová Rychnavská, Manon Slot, Sicco Scherjon, Nicolaus Kröger, Arnold Ganser, Eva M. Weissinger, Els Goulmy, Lothar Hambach

**Affiliations:** 1 Department of Immunohematology and Blood Transfusion, Leiden University Medical Center, Leiden, The Netherlands; 2 Sanquin Blood Supply Foundation, Division of Research, Department of Transfusion Medicine, Leiden, The Netherlands; 3 Department of Hematology, Hemostasis, Oncology and Stem Cell Transplantation, Hannover Medical School, Hannover, Germany; 4 Department of Obstetrics, Leiden University Medical Center, Leiden, The Netherlands; 5 Department of Bone Marrow Transplantation, University Medical Center Hamburg-Eppendorf, Hamburg, Germany; German Red Cross Blood Service Frankfurt, GERMANY

## Abstract

Persistent complete donor chimerism is an important clinical indicator for remissions of hematological malignancies after HLA-matched allogeneic stem cell transplantation (SCT). However, the mechanisms mediating the persistence of complete donor chimerism are poorly understood. The frequent coincidence of complete donor chimerism with graft-versus-leukemia effects and graft-versus-host disease suggests that immune responses against minor histocompatibility antigens (mHags) are playing an important role in suppressing the host hematopoiesis after allogeneic SCT. Here, we investigated a possible relationship between donor immune responses against the hematopoiesis-restricted mHag HA-1 and the long-term kinetics of host hematopoietic chimerism in a cohort of 10 patients after allogeneic HLA-matched, HA-1 mismatched SCT. Functional HA-1 specific CTLs (HA-1 CTLs) were detectable in 6/10 patients lysing host-type hematopoietic cells in vitro. Presence of HA-1 CTLs in the peripheral blood coincided with low host hematopoiesis levels quantified by highly sensitive mHag specific PCR. Additionally, co-incubation of host type CD34^+^ cells with HA-1 CTLs isolated after allogeneic SCT prevented progenitor and cobblestone area forming cell growth in vitro and human hematopoietic engraftment in immunodeficient mice. Conversely, absence or loss of HA-1 CTLs mostly coincided with high host hematopoiesis levels and/or relapse. In summary, in this first study, presence of HA-1 CTLs paralleled low host hematopoiesis levels. This coincidence might be supported by the capacity of HA-1 CTLs isolated after allogeneic SCT to specifically eliminate host type hematopoietic stem/progenitor cells. Additional studies involving multiple mismatched mHags in more patients are required to confirm this novel characteristic of mHag CTLs as factor for the persistence of complete donor chimerism and leukemia remission after allogeneic SCT.

## Introduction

Leukemia relapse is the main cause for mortality after HLA-matched allogeneic stem cell transplantation (SCT) [1,2]. The overall relapse risk after allogeneic SCT is strongly influenced by the intrinsic properties of the leukemia, such as karyotype or somatic gene mutations [3,4]. Complete donor chimerism is an important clinical indicator for remissions of hematological malignancies in individual patients after allogeneic SCT [5,6]. Conversely, rapid expansion of the host hematopoietic cell compartment is strongly associated with relapse [5,6]. The impact of the transplanted donor immune system on complete donor chimerism is well documented by the positive effects of donor lymphocyte infusions (DLI) [7,8] and by the negative effects of T-cell depletion [9]. Moreover, Graft-versus-host disease (GvHD) is frequently associated with the conversion to complete donor chimerism [5,6]. These observations suggest that T-cell responses regulating donor chimerism are directed against the same targets as those mediating graft-versus-leukemia (GvL) effects and GvHD, namely minor histocompatibility antigens (mHags).

MHags are highly immunogenic polymorphic peptides derived from cellular proteins and presented in HLA-molecules [10]. Most mHags exist in two alleles based on a single nucleotide polymorphism in the encoding gene. Mostly only one allele forms immunogenic T-cell epitopes leading to strong immune responses after HLA-matched but mHag mismatched SCT [10]. MHags show a differential tissue distribution, which allows a separation of GvL effects from GvHD [11]. Namely, ubiquitously expressed mHags have been identified as the prime in situ targets of GvHD [12]. Consequently, mismatched hematopoiesis-restricted mHags might be therapeutically exploited to evoke strong GvL effects with low risk of GvHD [10]. The hematopoiesis-restricted mHag HA-1 appears particularly suitable for immunotherapeutic purposes since it is highly immunogenic [13] and its expression is shared by virtually all hematopoietic cells including normal [14] and leukemic progenitors [15,16], lymphoma [17] and multiple myeloma cells [18]. Only the HA-1^H^ but not the HA-1^R^ allele forms immunogenic T-cell epitopes (in HLA-A2 and –B60) [10]. Thus, HLA-matched/HA-1 mismatched SCT can evoke strong T-cell responses of the HA-1^RR^ donor against hematopoietic cells of the HA-1^HR/HH^ patient. The in vivo relevance of HA-1 in the GvL effect is documented by several observations. First, HA-1 specific cytotoxic T-lymphocytes (HA-1 CTLs) are capable of eradicating human leukemia cells in immunodeficient mice [19]. Second, HA-1 CTLs emerging after donor-lymphocyte infusions (DLI) subsequent to T-cell depleted SCT coincide with molecular remissions of relapsed CML and mutiple myeloma [20]. Finally, the leukemia relapse risk is lower in patients with GvHD subsequent to HA-1 mismatched compared to HA-1 matched allogeneic SCT [21–23].

Since HA-1 CTLs isolated after allogeneic SCT eliminate both malignant and non-malignant host hematopoietic cells [14–18,20] in vitro and in vivo, we hypothesized that a sustained immune reconstitution with HA-1 CTLs is associated with a persistently complete donor chimerism after allogeneic SCT. Here, we studied for the first time the relationship between the long-term HA-1 specific immune response and host hematopoiesis levels after T-cell replete HLA-matched, HA-1 mismatched SCT.

## Materials and Methods

### Subjects studied

All HLA-A2^+^ patients receiving an allogeneic 10/10 HLA-matched (on a high-resolution level for exon 2+3 for HLA-A, B, C and for exon 2 for HLA-DRB1 and -DQB1 according to the current European Federation for Immunogenetics guidelines) SC graft between May 2005 and August 2006 at the Hannover Medical School were typed for the mHags HA-1 and HA-2 [24]. Patients included in the study were mismatched for the mHag HA-1 (donor HA-1^RR^, patient HA-1^H^) and compatible for the mHag HA-2 (donor and patient HA-2^V^) with their donors. Patients were treated according to SCT protocols approved by the Institutional Review Board of the Hannover Medical School. Patients and donors gave written informed consent in accordance with the declaration of Helsinki. Details are provided in Table 1 and S1 Appendix. Blood samples were obtained in EDTA, PBMCs were isolated by ficoll gradient, frozen and stored in liquid nitrogen. Analysis was performed with approval of the Institutional Review Boards of the Leiden University Medical Center (CME09/085/NV) and of the Hannover Medical School (1062–2011).

**Table 1:** Patient characteristics.

NO	Disease	Age	Disease status at SCT	Conditioning	Gender D → P	Sib	GvHD prophy-laxis	Graft	CD34+ cells (10^6^/kg)	Neutro >500/ul on day	Outcome (cause of death)	Maximum grade GvHD	DLI	Lowest host chimerism (%)
1	AML	41	CR2	FLAMSA/TBI/Cy/ATG	F → F	No	CsA/MMF	PBSC	6.2	26	Relapse d149	aG: none	Day 142	0.05
											Death d391 (relapse)	cG: none		
2	PMF	58	Dupriez score intermed.	Flu/Bu/ATG	F → F	Yes	CsA/MTX	PBSC	3.7	31	CR	aG: none	none	0.006 *
											Alive > d1701	cG: none		
3	ALL	18	CR1	TBI/VP-16/Thy	F → M	Yes	CSA/Pred	PBSC	5.5	15	CR	aG: IV	None	0.002
											Death d275 (fungal infection)	cG: extensive		
4	AML	39	PR1	FLAMSA/TBI/Cy/ATG	F → M	Yes	CsA/MMF	PBSC	6.7	28	CR	aG: none	None	0.002
											Alive >d1651	cG: extensive		
5	AML	59	PR1	FLAMSA/TBI/Cy/ATG	F → M	No	CsA/MMF	PBSC	7.15	16	Relapse d453	aG: II	Day 486	0.005
											Death d654 (MOF)	cG: extensive	Day 528	
													Day 552	
6	sAML	51	CR2	FLAMSA/TBI/Cy/ATG	M → M	No	CsA/MMF	PBSC	4.5	20	Relapse d503	aG: I	Day 554	0.08
											Death d984 (relapse)	cG: none	Day 582	
7	CLL	48	PR2	TBI/Flu/Cy/Cam	M F	No	CsA	BM	1.45	17	PD	aG: II	None	0.8
											Death d290 (sepsis)	cG: extensive		
8	SAA	37	PD	TBI/Flu/Cy	F → F	Yes	CsA	BM	1.9	22	Well	aG: none	None	1.7
											Alive>d1319	cG: none		
9	CLL	41	PR2	Flu/Cy	M → M	Yes	CsA/MTX	PBSC	7.4	15	PD	aG: none	Day 237	15.18
											Alive >d1306	cG: none	Day 266	
													Day 306	
10	PMF	46	Dupriez score intermed.	Flu/Bu/ATG	M → M	No	CsA/MTX	PBSC	21.6	24	CR	aG: none	Day 834	0.005 *
											Alive >d1763	cG: extensive	Day 862	
													Day 889	

**Abbreviations**: AML, acute myeloid leukemia; sAML, secondary AML; ALL, acute lymphoblastic leukemia; PMF, primary myelofibrosis; CLL: chronic lymphoblastic leukemia; SAA, severe aplastic anemia; Sib: matched sibling donor; PR: partial remission; CR: complete remission; PD: progressive disease; Dupriez score for the prognosis of PMF [29]; Bu, Busulphan; Cy, Cyclophosphamide; Flu, Fludarabin; TBI, Total Body Irradiation; VP-16, Etoposide; the FLAMSA protocol consisted of Fludarabine, Cytosine-Arabinoside and Amsacrine; D, donor; P, patient; M, male; F, female; MMF, Mycophenolate Mofetil; MTX, Methotrexate; CsA, Cyclosporine A; Pred, prednisolone; ATG, Anti-Thymocyte Globulin; Thy, Thymoglobuline; Cam, Campath; PBSC, peripheral blood stem cells; BM, bone marrow; MOF: multi organ failure; aG: acute GvHD graded according Glucksberg score [30]; cG: chronic GvHD graded according Seattle classification [31];

* lower detection limit of the used HA-1^H^ based chimerism assay: 0.01%

### Monitoring of HA-1 CTLs

Allophycocyanin (APC)-conjugated HA-1^A2^ and HA-2^A2^ tetramers were generated as described [25]. PBMCs were thawed and incubated overnight in 20% human serum (HS) in IMDM (Biowhittaker, Verviers, Belgium) at 37°C, 5% CO2. Then, PBMCs were stained with HA-1^A2^ or HA-2^A2^ tetramer dilutions, CD3-FITC and CD8-PE (Becton Dickinson) antibodies and propidium iodide (PI) as described [25] and analysed with a FACS Calibur (Becton Dickinson) flow cytometer. Lymphocytes were gated in a forward/sideward scatter, viable PI^-^/CD3^+^ T-cells were selected and presented in a plot with CD8 and HA-1^A2^ (or HA-2^A2^) tetramer. HA-2^A2^ tetramers were used to determine background staining in every sample. Absolute CD8^+^/HA-1^A2^ tetramer^pos^ cells/ml peripheral blood were calculated as follows: white blood cells/ml x %lymphocytes x (%CD3^+^/CD8^+^/HA-1^A2^ tetramer^pos^ cells in the PI^-^ lymphocyte gate – % CD3^+^/CD8^+^/HA-2^A2^ tetramer^pos^ cells in the PI^-^ lymphocyte gate).

### Isolation of mHag-specific CTL clones

HA-1^A2^ tetramer^pos^ CD8^+^ cells in PBMCs were sorted at one cell per well by using a FACSVantage cell sorter (Becton Dickinson) into a 96 well plate containing 10% HS in IMDM, irradiated allogeneic PBMCs, EBV LCLs, 1% phytohemagglutinin and 120 IU /ml IL-2 (Chiron, Amsterdam, The Netherlands). CTLs were cultured until sufficient HA-1 CTLs could be harvested for functional assays.

### Chromium release assay

Cytotoxicity was tested in a standard 4h chromium release assay as described [26]. In short, 2500 ^51^Cr-labeled target cells were incubated with serial dilutions of effector CTLs for 4 hours; supernatants were harvested for gamma counting: % specific lysis = (experimental release—spontaneous release)/(maximal release—spontaneous release) x 100%.

### Quantification of host chimerism

Detection of Y-chromosome^pos^ and HA-1^H^ chimeric cells was performed on PBMCs as described earlier [27]. In short, the DNA was isolated with the QIAamp DNA blood minikit (Qiagen, Leusden, The Netherlands). Y-chromosome specific chimerism was analyzed using a one-step real-time PCR protocol. A second PCR reaction detecting the human Hematopoietic Cell Kinase gene (HCK) was carried out in parallel to standardize the data. Detection of HA-1^H^ chimeric cells was performed using a nested PCR protocol. Amplification data were collected and analyzed with the MyiQ^TM^ Single-Color real-time PCR detection system (Bio-Rad Laboratories, Veenendaal, The Netherlands). The data are expressed as normalized fold expression, which was calculated according to the corresponding HCK levels. All samples were tested in at least two separate tests. A titration series of a mHag^pos^ EBV LCL Cell Line [11] diluted in mHag^neg^ EBV-LCL [11] was used as reference for linear regression analysis.

### CD34^+^ and CD133^+^ cell purification

Mononuclear cells were isolated from CB (partially kindly provided by Gesine Koegler, Institute for Transplantation Diagnostics and Cell Therapeutics, University of Duesseldorf Medical School, Duesseldorf) or BM using a ficoll density gradient. CD34^+^ or CD133^+^ cells were isolated by magnetic cell separation using the direct CD34^+^ or CD133^+^ progenitor cell isolation kit (Miltenyi Biotec GmbH, Bergisch Gladbach, Germany), checked for purity by flow cytometry and frozen at −80°C until use. The CD34^-^ or CD133^-^ fraction was used for HA-1 typing.

### In vitro progenitor and stem cell assays

5000 umbilical cord blood (UCB) or bone marrow (BM) derived CD34^+^ or CD133^+^ cells were co-incubated overnight with irradiated (3000 rad) CTLs (CMV CTL clone 5D5 [19], HA-1 CTL clone 3HA15 [28] or allo-A2 CTL clone MBM13 (kindly provided by Prof. Fred Falkenburg, LUMC, The Netherlands)) at an effector to target ratio of 7:1 in a 96 round bottom plate in 10% HS in IMDM. In total, 6 wells per condition were plated. The next day, cells were washed twice with CAFC medium (IMDM supplemented with 3.2% inactivated FCS, 3.2% HS, 2.3 mM glutamine (Gibco, Breda, The Netherlands), 3x10^2^ U/ml penicillin (Bio-Whittaker) and 3x10^2^ μg/ml streptomycin (Bio-Whittaker), 7.2x10^−3^mM hydrocortisone (Sigma, Zwijndrecht, The Netherlands) and 7.2 mM β-mercapto-ethanol (Sigma)). Viable CD34^+^ cells were quantitatively determined by flow cytometry with CD45-FITC, CD34-PE, 7AAD and Flow-Count fluorospheres (all Beckman Coulter, Mijdrecht, The Netherlands). Cells of 1 well per condition were subjected to a liquid human progenitor cells (HPC) assay and cells of 4–5 wells per condition were subjected to a cobblestone area-forming cell (CAFC) assay.

### Liquid HPC and HALO assays

HPC assays were performed as described by the manufacturer (StemCell Technologies Inc., Grenoble, France). In short, 1000 BM- or 200 UCB-derived CD34^+^ or CD133^+^ cells (supplemented with CTLs or not) were cultured in 1.1 ml Methocult dispensed in 35-mm dishes (Greiner, Alphen a/d Rijn, The Netherlands) for 14 days at 37°C, 5% CO_2_. The number of erythroid, granulocyte and monocyte colonies was expressed as number of colonies formed per plate using an inverted light microscope. The HALO-96 human assay was used to detect human progenitor cell proliferation in mouse BM as described by the manufacturer (HemoGenix, Colorado, USA). Briefly, 1,25x10^5^ BM WBC/well were plated in a 96-wells plate and incubated at 37°C, 5% CO_2_ for 6 days with HPC specific cytokine mix (HemoGenix). Intracellular ATP content served as substrate for a luciferin/luciferase reaction was measured as relative light units (RLU) (Centro LB 960 luminometer, Berthold Technologies, Vilvoorde, Belgium) and was determined exactly based on an ATP standard curve included into the assay.

### Cobblestone area forming cell assay

Irradiated (500 rad) 2.7x10^4^x10^4^ NIH3T3 mouse fibroblasts in IMDM supplemented with 10% FCS were added to a 96 well flat bottom plate pre-coated with 1% gelatin. The next day, fibroblasts were overlain with the CD34^+^ cells obtained from the overnight incubation with CTLs or medium. Subsequently, the cells were cultured at 37°C in 5% CO_2_; medium was replenished weekly by exchanging half of the medium with fresh CAFC medium. After 5 weeks, wells were scored positive if at least one phase-dark hematopoietic clone (a cobblestone area of at least 4 cells) was observed.

### NOD/SCID repopulation experiments

8–10 weeks old female NOD.CB17-prkdc<scid>/J (NOD/SCID) mice (Charles River, France) were used after ethical approval by the Leiden University Medical Center review board on animal studies (Permit Number: 08189). 24 hours after sublethal irradiation with 3.5 Gy, NOD/SCID mice received a tail injection with 2x10^5^ CD34^+^ cells pre-incubated overnight in a sterile FACS tube with irradiated CTLs (5D5, 3HA15 and MBM13) at an effector to target ratio of 7:1. Three UCBs were used with 3–4 mice per group for each UCB. Mice were sacrificed after 14 and 16 weeks. Cell suspensions (BM and spleen) and peripheral blood were prepared and analyzed by flow cytometry as described [19].

### Statistical Analysis

Statistical analysis was performed using SPSS 16.0 (SPSS Inc., Chicago, IL, USA). A p-value < 0.05 was considered statistically significant.

## Results

### Kinetics of HA-1 CTL responses after allogeneic SCT

Ten patients transplanted with SC grafts of HLA-matched, HA-1 mismatched donors were included in this study. The patient characteristics are shown in Table 1 and detailed clinical information is provided in S1 Appendix.

**Fig 1:**
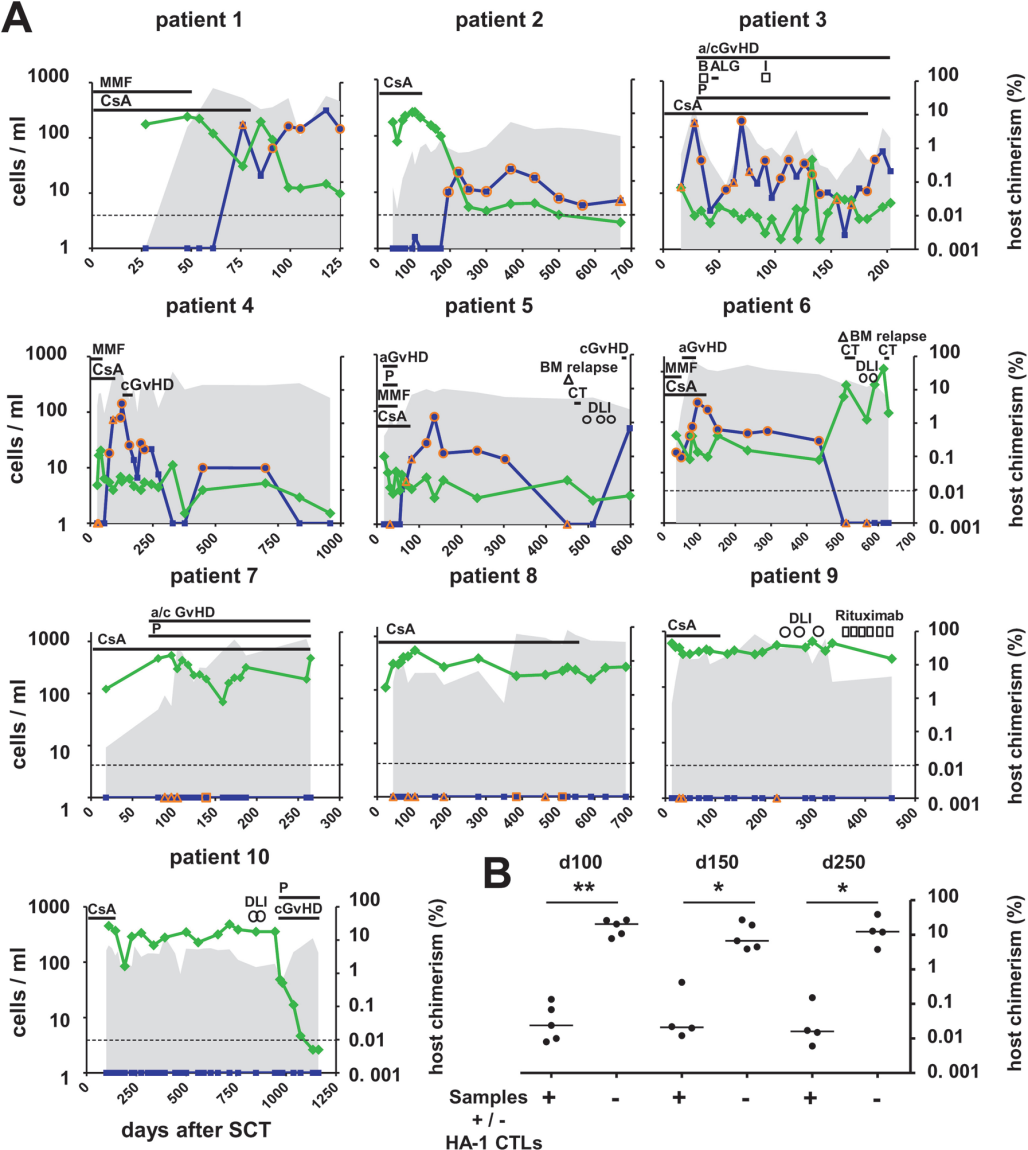
Kinetics of the donor HA-1 CTL response in relation to host chimerism in the peripheral blood. (A) Depicted are the results of 10 patients undergoing allogeneic HLA-matched, HA-1 mismatched SCT; x-axis: days after allogeneic SCT; left y-axis, blue squares and line: T-cells in absolute numbers per ml peripheral blood detected and calculated as described in Materials and Methods; open circles: samples from which HA-1 CTLs could be isolated capable of lysing Epstein-Barr virus/lymphoid cell lines (EBV LCLs) expressing the HA-1^H^ natural ligand; open triangles: samples with unsuccessful attempts to isolate HA-1^A2^ tetramer staining cells; shaded areas: CD8^+^ T-cells in 10^3^ cells per ml peripheral blood; right y-axis, green diamonds and line: host chimerism determined by highly sensitive mHag HA-1 (patient 1–2, 6–10) or H-Y (patient 3–5) specific quantitative PCR. Horizontal dotted line: lower detection limit for HA-1^H^ allele specific PCR: 1 host in 10^4^ donor cells; the lower detection limit for H-Y specific PCR is 1 host in 10^5^ donor cells; horizontal black bars indicate times of GVHD, immunosuppression (CsA, MMF, P, ALG, B and I) or CT, black open circles indicate DLI, black open triangles indicate relapse, black open squares indicate rituximab treatment, Abbreviations: CsA, cyclosporine A; MMF, mycophenolate mofetile; P, prednisolone; B, basiliximab; I, infliximab; ALG: anti-lymphocyte globulin; CT: chemotherapy. (B) Comparison of the host chimerism levels in samples with and without HA-1 CTLs collected on days 100, 150 and 250 (+/-27) after allogeneic SCT. The horizontal lines indicate the median values. Results from a two-tailed Mann-Whitney-U test are shown (*p<0.05).

The median observation time post transplant was 613 days (range 125–1162). Presence of HA-1 CTLs was analyzed in donor PBMCs obtained prior to SC collection and in all available patient PBMC samples before and after allogeneic SCT. None of 8 evaluable SC donors showed HA-1 CTLs in the peripheral blood before SCT. Post-transplant patient’s samples collected at 18 (median; range 9–36) post-transplant time points per patient with a median interval of 15 days (median; range 5–250) were analyzed. HA-1 CTLs were detectable in 6/10 patients (patients 1–6) after SCT with frequencies between 0.02% and 0.14% of the viable CD3^+^/CD8^+^ T-cells. HA-1 CTLs were first detected 72 days (median; range 28–195) after allogeneic SCT (Fig. 1). Increment of HA-1 CTLs was detectable 26 days (median; range 11–70) after discontinuation of CsA, MMF or prednisolone (patient 1,2, 4–6), 19 days (median; range 0–69) after onset of acute or chronic GvHD (patients 3–6) and 46 days after the last DLI (patient 5). Additional HA-1 CTL peaks were found during the course of GvHD (patient 3). HA-1 CTLs were observed until day 516 (median; range 125–699) after SCT. In 2/6 HA-1 CTL^pos^ patients (patient 5 and 6), HA-1 CTLs disappeared during the observation period on day 450 and 503, respectively.

### Relationship between HA-1 CTLs and host chimerism after allogeneic SCT

Commonly applied methods to identify complete donor chimerism as marker of leukemia remission have a sensitivity of detecting 1 host in 10^2^ donor cells [5]. In this study, host chimerism was measured by highly sensitive mHag allele specific quantitative PCR which can detect 1 host in 10^4^ (HA-1 based) or 10^5^ (H-Y based) donor cells. HA-1 specific quantitative PCR was applied in patients 1–2 and 6–10. In 3 male patients with female donors (patients 3–5), host chimerism was determined by H-Y specific quantitative PCR. A qualitative comparison of the course of HA-1^A2^ tetramer^pos^ cells with the course of host chimerism in individual patients revealed that emergence of HA-1 CTLs paralleled a rapid decline of host chimerism (patient 1 and 2; Fig. 1A). Presence of HA-1^A2^ tetramer^pos^ cells coincided with host chimerism levels < 1% (patient 1–6; Fig. 1A) mostly just above the detection limit (patient 2–5). Loss of HA-1 CTLs was followed by leukemia relapse (patient 5 and 6; Fig. 1A) and rapid expansion of host cells in the peripheral blood (patient 6; Fig. 1A). The four HA-1 CTL^neg^ patients (patient 7–10) had host chimerism levels >1% persistently almost throughout an observation period of up to 900 days (patient 7–10; Fig. 1A). One HA-1 CTL^neg^ patient with persistently high host chimerism levels > 1% showed a rapid decline of host chimerism to undetectable levels after day 900 subsequent to two DLIs (patient 10; Fig. 1A). Comparison of all samples with both available HA-1^A2^ tetramer and host chimerism data revealed that 63/66 HA-1 CTL^pos^ samples had host chimerism levels <1%, while only 23/105 HA-1 CTL^neg^ samples had host chimerism levels <1% (S1 Fig.). Next, we compared host chimerism levels in samples with and without HA-1 CTLs only at post-transplant time points with **≥** 4 samples/group (i.e. on day 100, 150 and 250 +/-27) for proper analysis of independent samples at small sample size. Host chimerism values were significantly lower in samples with HA-1 CTLs compared to samples without HA-1 CTLs (p<0.05, two-tailed Mann-Whitney-U-test; Fig. 1B). Overall, presence of HA-1 CTLs coincided with low host chimerism levels in our cohort.

### Isolation and functional characterization of HA-1 CTLs

**Fig 2:**
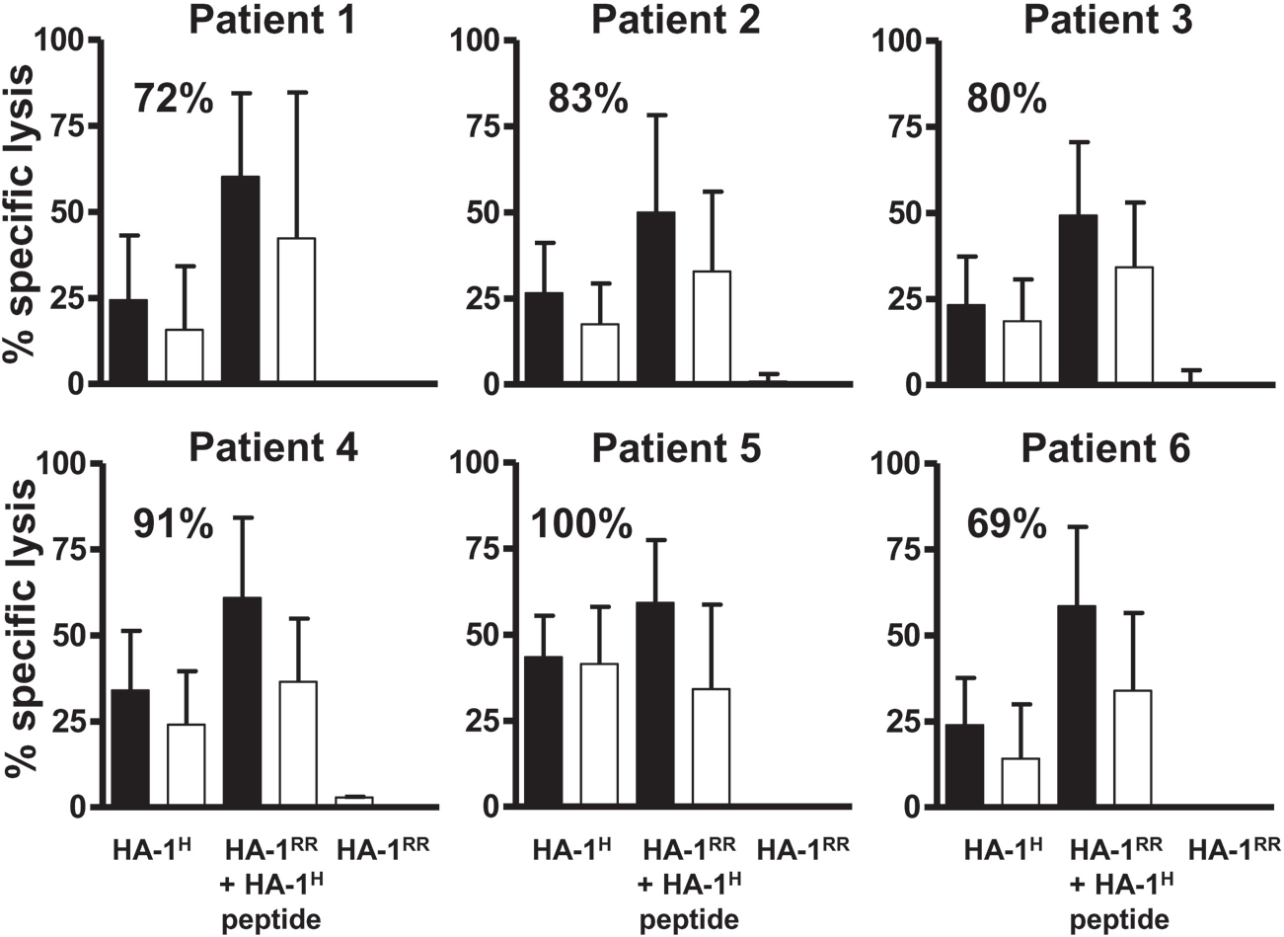
Cytotoxicity of isolated HA-1 CTLs. HA-1 CTL clones were isolated from peripheral blood of patients 1–6 at multiple time-points and expanded in vitro. Median 13 (range: 8–25) HA-1 CTL clones per patient were tested in a 4h ^51^Cr-release assay for HA-1 specific recognition of target cells in vitro. Left upper corner: percentage of HA-1 CTL clones lysing HA-1^H^ naturally expressing EBV LCLs; x-axis: HA-1^H^ naturally expressing EBV LCLs, HA-1^RR^ EBV LCLs loaded with HA-1^H^ peptide, HA-1^RR^ EBV LCLs, E/T ratio: back: 10:1, white: 1:1; y-axis: % specific lysis.

HA-1 CTLs were isolated from 57 of 71 HA-1^A2^ tetramer^pos^ PBMC samples after allogeneic SCT by single-cell sorting and expansion from patients 1–6. Sixty (median; range 34–142) HA-1 CTL clones per patient were isolated and expanded in vitro. Thirteen (median; range 8–25) HA-1 CTL clones per patient were tested in a ^51^Cr-release assay for cytotoxicity against HA-1^H^ (natural ligand of host type), HA-1^RR^ (natural ligand of donor type) and HA-1^H^ peptide loaded HA-1^RR^ target cells. From additional 15 (median; range 7–18) HA-1 CTL clones per patient, sufficient cell numbers were only available for testing lysis of HA-1^H^ natural ligand and of HA-1^H^ peptide loaded HA-1^RR^ target cells. In summary, 82% (median; range: 69–100%) of all analyzed HA-1 CTL clones recognized target cells expressing the HA-1^H^ natural ligand and 2% (median; range: 0–25%) of the HA-1 CTL clones recognized only HA-1^H^ peptide loaded target cells. None of the HA-1 CTL clones tested against target cells expressing the HA-1^RR^ of the donor showed cytotoxicity (Fig. 2). All clinical samples wherein natural HA-1^H^ ligand specific HA-1 CTL clones were successfully isolated are indicated in Fig. 1 (open circles). PBMCs without detectable HA-1^A2^ tetramer^pos^ cells from 6 patients (patient 4–9) were used as control for the validity of the HA-1^A2^ tetramer staining to detect functional HA-1 CTLs (open triangles). None of the HA-1^A2^ tetramer^neg^ PBMC samples did contain HA-1 CTL clones recognizing the natural HA-1^H^ ligand. Overall, 46 of the 57 tested HA-1^A2^ tetramer^pos^ PBMCs samples contained HA-1 CTLs specifically lysing hematopoietic cells expressing the HA-1^H^ ligand of the host.

### HA-1^H^ CTLs eliminate human hematopoietic stem/progenitor cells in vitro

**Fig 3:**
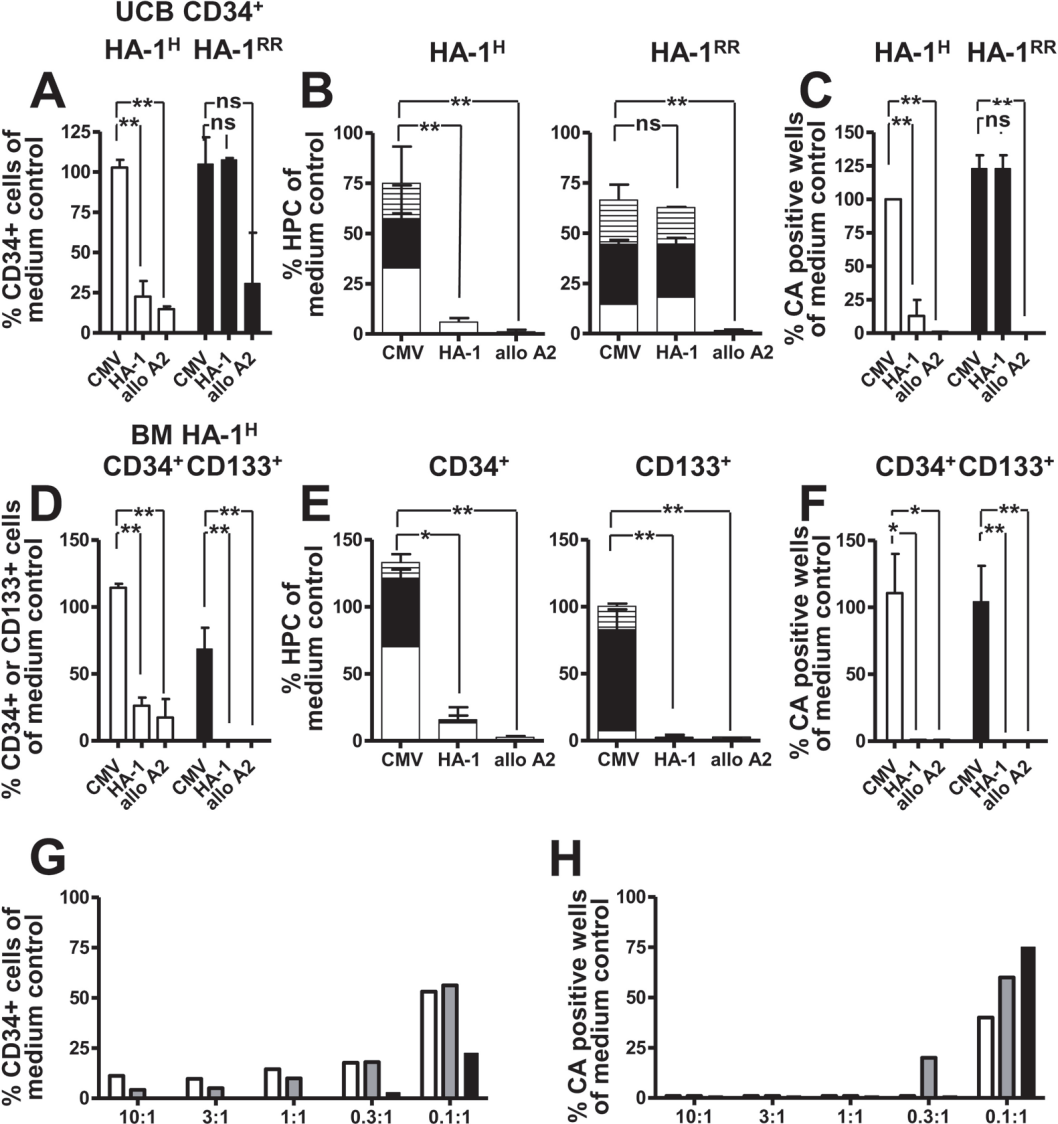
HA-1^H^ specific elimination of human umbilical cord blood and bone marrow derived hematopoietic progenitor/stem cells by HA-1 CTLs studied in vitro. (A-F) Umbilical cord blood (UCB) derived CD34+ and bone marrow (BM) derived CD34^+^ and CD133^+^ cells from HLA-A2/HA-1^H^ or HLA-A2/HA-1^RR^ donors were incubated with medium, an irradiated control CMV CTL clone, an HA-1 CTL clone or an anti-HLA-A2 specific CTL clone overnight. Data are mean +/- Standard deviation of independent experiments with 3 HA-1^H^ and 2 HA-1^RR^ donors, respectively (*p<0,05; **p<0.01, ns = not significant using a two-tailed Student’s t-test). The results of the medium control were set as 100%. (A, D) % viable CD34^+^ or CD133^+^ cells (duplicate sample per donor) compared to the medium control directly after over-night co-incubation with CTLs. (B, E) % colony forming cells (single sample per donor) compared to the medium control as determined after 14 days; CFU-macrophage (CFU-M; striped bar), CFU-Granulocyte (CFU-G; black bar), blast forming unit (BFU-E; white bar). (C, F) % of cobblestone area positive wells (4–5 samples per donor) in a CAFC assay compared to the medium control as determined after 5 weeks. (G-H) An HA-1 CTL clone was titrated to BM derived CD34+ cells (white bars), UCB derived CD34+ cells (grey bars) and to BM derived CD133^+^ cells (black bars) from HLA-A2/HA-1^H^ donors and co-incubated over night. x-axis: HA-1 CTL to CD34 or CD133 cell ratio. Y-axis: % viable CD34^+^ or CD133+ cells (duplicate sample per donor) compared to the medium control (G) and % of cobblestone area positive wells in a CAFC assay (4–5 samples per donor) compared to the medium control as determined after 5 weeks (H).

Next, the capacity of HA-1 CTLs to eliminate human hematopoietic stem/progenitor cells (HSPCs) in vitro was investigated. CD34^+^ cells were isolated from HLA-A2^+^/HA-1^H^ or HA-1^RR^ umbilical cord blood (UCBs) and co-incubated overnight with medium, a control CMV CTL clone 5D5, the well defined HA-1 CTL clone 3HA15 isolated after allogeneic SCT [28] and an allo HLA-A2 specific CTL clone MBM13. CD34+ or CD133+ cells were quantified directly after overnight co-incubation, progenitor cells were determined in standard human progenitor cell (HPC) assays after 14 days and presence of stem cells was analyzed in cobblestone area forming cell (CAFC) assays after 5 weeks. Co-incubation of HA-1^RR^ (i.e. donor type) CD34^+^ cells with the HA-1 CTL clone resulted in no significant differences in viable CD34^+^ cell numbers, HPC colonies and CAFC colonies compared to the medium and cytomegalovirus (CMV) CTL control (Fig. 3A-C). In contrast, co-incubation of HA-1^H^ (i.e. patient type) CD34^+^ cells with the HA-1 CTL clone resulted in a considerable reduction of viable CD34^+^ cells in the flow cytometry, of particularly CFU-G (granulocyte) and –M (monocyte/macrophage) growth in the HPC assays and of the colony formation in the CAFC assay compared to the medium and CMV CTL control (p<0.05; two-tailed Student’s t-test; Fig. 3A-C). Similar results were obtained after co-incubation of bone marrow (BM) derived HA-1^H^ CD34^+^ and CD133^+^ cells with HA-1 CTLs (Fig. 3D-F). These data indicate that HA-1 CTLs can eliminate UCB and BM derived HA-1^H^ HSPC and that HA-1 CTL mediated HSPC killing is specific for the presence of HA-1^H^. In all experiments, the allo-HLA-A2 specific control CTLs killed CD34+ or CD133+ cells, suppressed HPC growth and entirely prevented colony formation in CAFC assays compared to the medium and CMV CTL control (Fig. 3A-F). Finally, the HA-1 CTL clone was titrated to human UCB derived CD34^+^ cells, BM derived CD34^+^ and BM derived CD133^+^ cells from HLA-A2^+^/HA-1^H^ donors. Co-incubation with HA-1 CTLs resulted in a comparable reduction of CD34^+^ (from UCB and BM) and CD133^+^ cells in the flow cytometry and a comparable suppression of CAFC colonies (Fig. 3G,H). The minimum required effector to target cell ratio ensuring complete suppression of CAFC colonies was 0.3–1:1. In summary, these data suggest that HA-1 is expressed on human HSPCs and functions as target for HA-1 specific lysis.

### HA-1^H^ is expressed on human NOD/SCID mice repopulating hematopoietic cells

**Fig 4:**
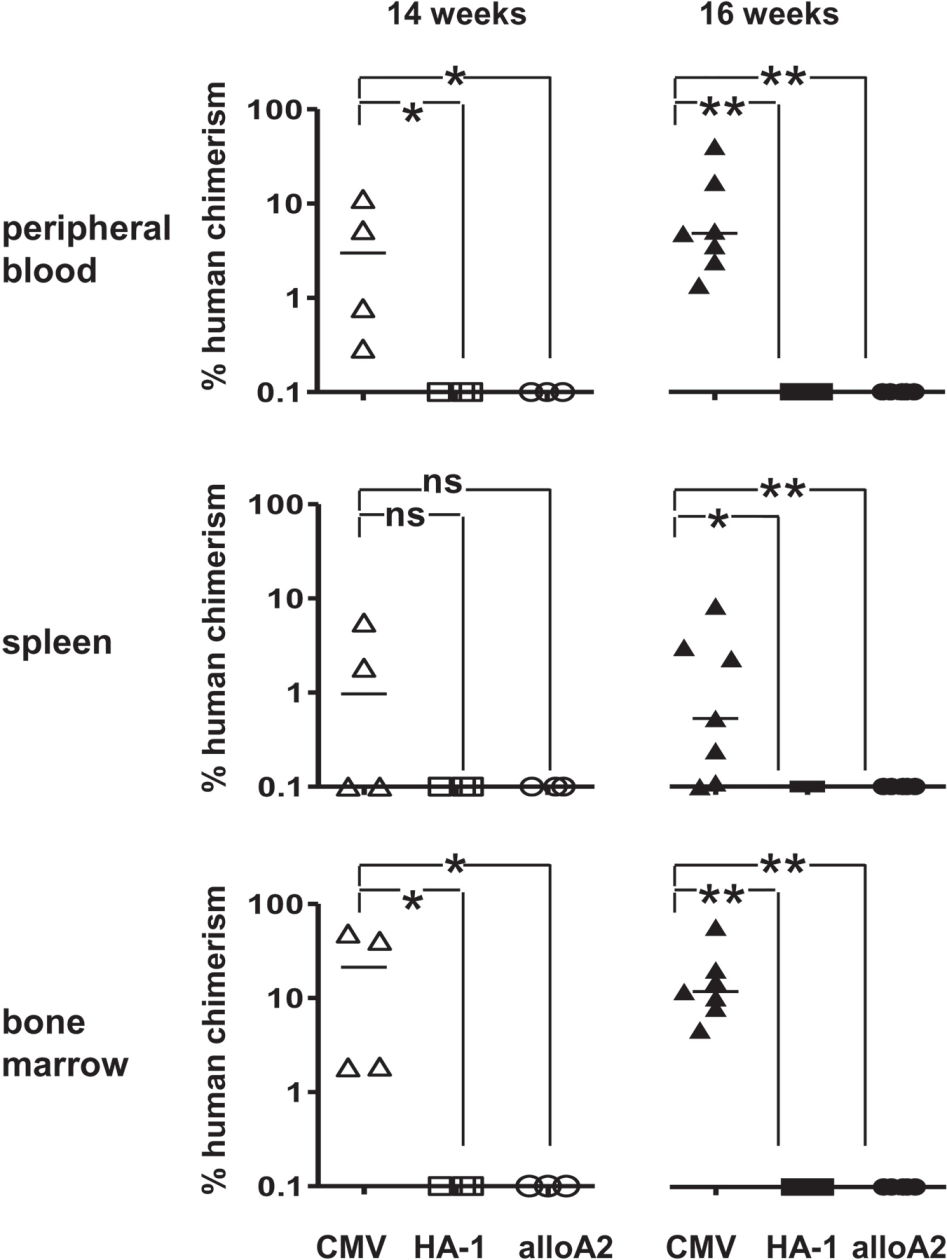
Elimination of NOD/SCID mouse repopulating human progenitor/stem cells by HA-1 CTLs. UCB derived CD34^+^ cells from three different HLA-A2/HA-1^H^ donors were incubated with an irradiated control CMV CTL clone (triangles), HA-1 CTL clone (boxes) or anti-HLA-A2 CTL clone (circles) and transplanted into sublethally irradiated NOD/SCID mice. After 14–16 weeks, mice were sacrificed. Depicted is the % human hematopoietic chimerism in the peripheral blood, spleen and bone marrow of the individual mice after 14 (white symbols; n = 4 mice per group) and 16 (black symbols; n = 3–4 mice per group and donor) weeks in three independent experiments. The bar represents the mean of the group. Results from unpaired Mann-Whitney U statistical analyses are shown (*p<0,05; **p<0,01; ns = not significant).

Next, we investigated whether HA-1 CTLs can eliminate human non-obese diabetic / SCID (NOD/SCID) mouse repopulating hematopoietic cells. UCB derived HLA-A2^+^/HA-1^H^ CD34^+^ cells were co-incubated with control CMV CTLs, HA-1 CTLs and allo-HLA-A2 specific CTLs (3–4 mice per group) overnight prior to transplantation into NOD/SCID mice. Human hematopoietic engraftment was determined 14 or 16 weeks after transplantation in three independent experiments. All mice transplanted with CD34^+^ cells co-incubated with control CMV CTL showed considerable human CD45^+^ cell engraftment in the peripheral blood (pB, week 14–16: mean 8.5%, range 0.3–40.8), spleen (week 14–16: mean 2.0%, range 0.1–8.2) and BM (week 14–16: mean 20.1%, range 1.8–58.0) (Fig. 4). Sub-analysis of the human CD45^+^ cells in the bone marrow revealed presence of CD34^+^ (week 14–16: mean 3.7%, range 0.4–8.3), CD33^+^ (week 14–16: mean 4.1%, range 0.2–12.4) and CD19^+^ cells (week 14–16: mean 9.3%, range 2.2–20.5). In contrast, no human CD45^+^ cells were detectable in the peripheral blood, spleen or BM of mice transplanted with CD34+ cells co-incubated with HA-1 CTLs or with allo HLA-A2 specific CTLs (p<0.05; Mann-Whitney U-test at 14 weeks in pB and BM and at 16 weeks in pB, BM and spleen in comparison to CMV control mice; Fig. 4). Finally, BM cells of mice sacrificed after 16 weeks were further investigated for the presence of human cells capable of proliferation in progenitor cell assays. In the HALO assay, BM from mice transplanted with CMV CTL treated CD34^+^ cells showed human cell proliferation, while BM from mice transplanted with HA-1 CTL or with allo-HLA-A2 CTL treated CD34^+^ cells were negative for human cell proliferation (S2 Fig.). These data indicate that HA-1 is expressed on human hematopoietic HSPCs with the potential to engraft in NOD/SCID mice.

## Discussion

In this study we investigated for the first time the long term relationship between HA-1 specific immune responses and chimersim after T-cell replete allogeneic HLA-matched HA-1 mismatched SCT. Overall, HA-1 CTLs were detectable in 6/10 patients after T-cell replete HLA-matched, HA-1 mismatched SCT. However, HA-1 CTL numbers were unexpectedly low shortly (i.e. until median day 71) after allogeneic SCT considering the large numbers of HA-1 expressing host hematopoietic cells still present in some patients (patients 1, 2 and 6). The reasons for this observation are unclear. Very large quantity of antigen can suppress antigen specific immune responses [32]. Moreover, HA-1 is also expressed by (hematopoietic) non-antigen presenting cells, such as T-cells, which can be tolerogenic [33]. Thus, the relative overload of mHags presented in a partially tolerogenic context may hamper higher HA-1 CTL expansion shortly after allogeneic SCT. Increasing HA-1 CTLs were measurable at later time points (i.e. after median day 71) subsequent to discontinuation of immune suppression, GvHD or DLI. While HA-1 CTLs during GvHD [25] and subsequent to DLI [20] were described earlier, detection of HA-1 CTLs subsequent to the termination of immune suppression might have been missed in earlier studies because of the T-cell depleted SC grafts. Once detectable, HA-1 CTLs mostly persisted for months or years at low levels in our study. Thus, HA-1 CTLs can create a long lasting immunity after allogeneic SCT. However, for unknown reasons, the HA-1 CTL response terminated in two patients, on days 450 and 503, coinciding with leukemia relapse. It remains to be determined whether the loss of HA-1 CTLs might be associated with specific phenotypic changes, including e.g. differentiation or exhaustion markers that may allow a predication of the termination of the CTL response.

HA-1 CTLs were isolated and expanded for functional testing from almost all HA-1^A2^ tetramer^pos^ PBMC samples. This analysis revealed that HA-1 CTLs emerging after allogeneic SCT largely consist of high avidity, i.e. HA-1^H^ natural ligand specific CTLs lysing solely host type hematopoietic cells, while leaving donor hematopoietic cells intact. Four out of 10 patients analyzed did not show HA-1 CTLs in the entire post-transplant period regardless of the termination of immune suppression (patients 8–10), GvHD (patients 7,10) or DLI (patients 9,10). Thus, neither HA-1 mismatched SCT itself nor the described clinical events are sufficient to evoke detectable HA-1 CTLs. It remains unclear which factors determine presence or absence of HA-1 specific responses after allogeneic SCT. Firstly, pre-immunizations of healthy donors may have an impact [34], since both mHag sensitization and tolerization can occur through pregnancy in mothers and children [27,35,36]. This may involve responses in the mother against mHags inherited only from the father to the child and responses in the child against non-inherited mHags only present in the mother. However, HA-1 CTLs were not detectable in donor PBMC samples in our study, potentially due to the low precursor frequencies of mHag CTLs described earlier [27,35]. Further studies using larger donor cell numbers are required to understand whether HA-1 specific immunity is transferred from the donor to the patient. Additionally, CD4 T helper cell responses against HLA class II restricted mHag epitopes might enhance HA-1 CTL responses in some patients. However, since only few HLA class II restricted mHags have been identified to date [37,38] the impact of mHag specific CD4 T helper cell responses of HLA class I restricted mHags can hardly be determined. Finally, host derived professional antigen presenting cells (APCs) surviving conditioning may boost mHag CTL responses after SCT, which might be investigated by simultaneous monitoring of APCs and mHag CTLs [39]. Missing or disappearing mHag CTL responses in some patients in our study might also be explained by immune modulatory effects of leukemia cells suppressing the in vivo expansion of human mHag CTLs [40,41]. Of note, 2/4 HA-1 CTL negative patients (i.e. patient 7 and 9) were suffering from CLL which can induce T cell exhaustion e.g. via the expression of the ligand to the regulatory T cell surface PD-1 or of immunosuppressive metabolites [40,41]. Thus, leukemia relapse or persistence may induce loss of donor CTL’s, independently of their specific target. Nevertheless, absence of HA-1 CTLs was probably not resulting from a general immune paralysis in our study since CMV and EBV specific CTL responses monitored in separate experiments using fresh blood as described [42] were detectable in the HA-1 CTL negative patients 8 and 9 (S3 Fig.). The, in our opinion, most interesting and novel observation of the underlying study, is the apparent coincidence of the presence of HA-1 CTLs and the low to undetectable host chimerism levels (detected by highly sensitive quantitative PCR) in most samples throughout the observation period. Nevertheless, presence of HA-1 CTLs early after allogeneic SCT was not predictive for persistent remission. Namely, patients 1, 5 and 6 relapsed despite detectable levels of HA-1 CTLs early after allogeneic SCT. Notably, in the latter two patients samples were available until relapse. In these two patients, functional HA-1 CTLs disappeared later in time which is in accordance with our initial hypothesis that a sustained immune reconstitution with mHag CTLs is required for an ongoing control of the host hematopoiesis including the malignant cells. Accordingly, HA-1 CTL^neg^ patients showed persistently high host chimerism levels after allogeneic SCT. Evidently, if mHag CTLs play a role in the suppression of the host hematopoiesis after allogeneic SCT, this effect will not be restricted to solely HA-1. Namely, only 6.6% (sibling) to 12.0% (matched unrelated donor) of all allogeneic SCTs are performed with an HLA-A2/HA-1 mismatched donor graft [43]. Thus, immune responses against other known and unknown mismatched mHags need to be considered as well. This assumption is supported by the observation in patient 10 who converted to complete donor chimerism after DLIs without detectable HA-1 CTLs. Therefore, we also monitored the anti H-Y immune responses in male patients transplanted with a female donor (patients 3–5; data not shown). Interestingly, anti-HY responses were detectable only in patient 3, i.e. the only patient with grade IV GvHD in our study. This supports our conclusion that presence of a mHag mismatch alone is not sufficient to evoke a mHag specific immune response and that other factors such as GvHD and, potentially, donor pre-immunizations must be considered as well.

Highly sensitive quantitative PCR revealed the persistence of host chimeric cells throughout the post-transplant period in most of the patients in our cohort. Therefore, we raised the question whether HA-1 specific immune responses would be ultimately capable of eradicating the host hematopoiesis. This question becomes increasingly relevant with the use of reduced intensity conditioning where conversion to donor chimerism is considered to be more dependent on immunological factors than after myeloablative regimens [44]. We showed earlier that HA-1 CTLs can eliminate normal and malignant hematopoietic progenitor cells [14–16]. Obviously, adoptive HA-1 CTL transfer experiments in NOD/SCID mice would be the first choice to demonstrate the effect of HA-1 CTLs against human chimeric hematopoiesis in vivo. However, we previously showed that the in vivo efficacy of HA-1 CTLs – although highly suppressive against human chimeric leukemia cells – is limited in NOD/SCID mice by the short in vivo persistence of the HA-1 CTLs [19]. Therefore, we focused our studies on whether HA-1 is functionally expressed on normal host type hematopoietic HSPCs. HA-1 expression on normal hematopoietic stem cells was suggested earlier. This was based on the observation of host alloreactivity against HA-1 before SCT in patients rejecting their HA-1 mismatched donor graft [45]. In addition to previous reports [14–16], HSPCs were derived not only from BM but also from UCB and were further refined by surface expression of CD34 and CD133. Co-incubation of UCB and BM derived CD34^+^ or CD133^+^ stem cells with an HA-1 CTL clone established earlier from an allo-transplanted patient [28] abrogated not only HPC formation as described in our previous studies [14–16] but also CAFC colony formation in vitro. The effect of HA-1 CTLs on UCB derived CD34+ cells and BM derived CD34+ and CD133+ cells was comparable in the 5 week CAFC assays. Importantly, we showed for the first time that HA-1 CTLs prevented hematopoietic repopulation of NOD/SCID mice for at least 16 weeks post transplant indicating that also HSPC with engraftment potential are eliminated by HA-1 CTLs. Evidently, the residual malignant hematopoiesis also contributes to host chimerism in leukemia patients. The HLA-B8 restricted mHag UTY [46] and the HLA-B*2705 restricted mHag DDX3Y [47] are described to be expressed on leukemia stem cells. However, although HA-1 is expressed on virtually all hematological cancers [15–18], the formal proof of functional HA-1 expression by malignant stem cells is very cumbersome due to the controversial or still absent characterization of these cells in the various malignancies [48–50]. Overall, our data show that HA-1 CTLs can specifically destroy normal host type host hematopoietic cells up to the level of HSPCs. This may contribute to the observed coincidence of HA-1 CTLs with mostly very low host hematopoietic cell levels in our study. However, the persistence of host hematopoietic cells in all HA-1 CTL positive patients also indicates that the HA-1 specific immune response emerging after allogeneic SCT is not efficient enough to eradicate the host hematopoiesis. Therefore, we titrated HA-1 CTLs to purified CD34+ and CD133+ cells. Our data indicate that a HA-1 CTL to CD34+ or CD133+ cell ratio of at least 0.3–1:1 within in the stem cell niche would be necessary for the complete eradication of the host hematopoiesis. Considering the overall low frequency of HA-1 CTLs, it appears unlikely, that such high HA-1 CTL numbers at the stem cell niche are achieved in vivo. This leaves the risk of expansion of the normal and malignant host cell compartment at termination of the HA-1 CTLs response.

## Conclusions

Our data show in a small patient cohort for the first time that the immune reconstitution with functional CTLs against the mHag HA-1 coincides with low host hematopoiesis levels after T-cell replete HLA-matched, HA-1 mismatched allogeneic SCT. Despite the efficacy of HA-1 CTLs in specifically killing host type hematopoietic cells up to the level of HSPCs, the host hematopoiesis is not completely eradicated by HA-1 CTLs emerging after allogeneic SCT. Additional studies involving more patients and multiple mismatched mHags are required to confirm the postulated relevance of sustained mHag specific immune responses for the persistence of complete donor chimerism and leukemia remission after allogeneic SCT.

## Supporting Information

S1 Appendix Additional clinical information. (DOCX)

S1 Fig Comparison of the host chimerism levels in all samples with and without HA-1 CTLs. The horizontal lines indicate the median values.
(TIF)

S2 Fig Determination of human progenitor cell growth in the BM of NOD/SCID mice. BM of NOD/SCID mice transplanted with UCB derived CD34+ cells preincubated with CMV (black diamonds), HA-1 (red boxes) or alloA2 CTLs (green circles) was subjected 16 weeks after transplantation to an HALO progenitor cell assay and flowcytometry for human CD45. This assay determines intracellular ATP levels as measure for cell proliferation in response human cytokine stimuli. ATP is detected after 7 days of in vitro culture with bioluminscence in relative light units (RLU). Exact ATP concentrations were calculated based on the RLU in relation to a standard ATP curve. The %human CD45+ cells after 7 days of in vitro culture was determined for every HALO sample. X-axis: ATP concentration in μM; Y-axis: % human CD45% cells.
(TIF)

S3 Fig CMV and EBV CTLs after allogeneic SCT in patients 8 and 9. HLA-A2-restricted CMV (red line) and EBV CTLs (grey line) were detected by tetramer staining of fresh blood as described in [42]. Thus, data cannot be directly compared with the data performed on frozen samples presented in Fig. 1.
(TIF)

## References

[1] Horowitz M, Gale R, Sondel P, Goldman J, Kersey J, Kolb H, et al Graft-verus-leukemia reactions after bone-marrow transplantation. Blood. 1990; 75:555–562. 2297567

[2] Diaconescu R, Flowers CR, Storer B, Sorror ML, Maris MB, Maloney DG, et al Morbidity and mortality with nonmyeloablative compared with myeloablative conditioning before hematopoietic cell transplantation from HLA-matched related donors. Blood 2004; 104:1550–1558. 15150081 10.1182/blood-2004-03-0804

[3] Wagner K, Damm F, Thol F, Gohring G, Gorlich K, Heuser M, et al FLT3-internal tandem duplication and age are the major prognostic factors in patients with relapsed acute myeloid leukemia with normal karyotype. Haematologica. 2011; 96:681–686. 10.3324/haematol.2010.034074 21242187 PMC3084914

[4] Yoon JH, Kim HJ, Shin SH, Lee SE, Cho BS, Eom KS, et al Stratification of de novo adult acute myelogenous leukemia with adverse-risk karyotype: can we overcome the worse prognosis of adverse-risk group acute myelogenous leukemia with hematopoietic stem cell transplantation? Biol Blood Marrow Transplant. 2014; 20:80–88. 10.1016/j.bbmt.2013.10.015 24149098

[5] Kroger N, Bacher U, Bader P, Bottcher S, Borowitz MJ, Dreger P, et al NCI First International Workshop on the Biology, Prevention, and Treatment of Relapse after Allogeneic Hematopoietic Stem Cell Transplantation: report from the Committee on Disease-Specific Methods and Strategies for Monitoring Relapse following Allogeneic Stem Cell Transplantation. Part I: Methods, acute leukemias, and myelodysplastic syndromes. Biol Blood Marrow Transplant. 2010; 16:1187–1211. 10.1016/j.bbmt.2010.06.008 20558311 PMC7272718

[6] Kroger N, Bacher U, Bader P, Bottcher S, Borowitz MJ, Dreger P, et al NCI first international workshop on the biology, prevention, and treatment of relapse after allogeneic hematopoietic stem cell transplantation: report from the committee on disease-specific methods and strategies for monitoring relapse following allogeneic stem cell transplantation. part II: chronic leukemias, myeloproliferative neoplasms, and lymphoid malignancies. Biol Blood Marrow Transplant. 2010; 16:1325–1346. 10.1016/j.bbmt.2010.07.001 20637879

[7] Dey BR, McAfee S, Colby C, Sackstein R, Saidman S, Tarbell N, et al Impact of prophylactic donor leukocyte infusions on mixed chimerism, graft-versus-host disease, and antitumor response in patients with advanced hematologic malignancies treated with nonmyeloablative conditioning and allogeneic bone marrow transplantation. Biol Blood Marrow Transplant. 2003; 9:320–329. 12766882 10.1016/s1083-8791(03)00077-6

[8] Bethge WA, Hegenbart U, Stuart MJ, Storer BE, Maris MB, Flowers ME, et al Adoptive immunotherapy with donor lymphocyte infusions after allogeneic hematopoietic cell transplantation following nonmyeloablative conditioning. Blood. 2004; 103:790–795. 14525766 10.1182/blood-2003-07-2344

[9] Bretagne S, Vidaud M, Kuentz M, Cordonnier C, Henni T, Vinci G, et al Mixed blood chimerism in T cell-depleted bone marrow transplant recipients: evaluation using DNA polymorphisms. Blood. 1987; 70:1692–1695. 3311209

[10] Hambach L, Goulmy E. Immunotherapy of cancer through targeting of minor histocompatibility antigens. Curr Opin Immunol. 2005; 17:202–210. 15766682 10.1016/j.coi.2005.01.010

[11] de Bueger M, Bakker A, van Rood J, van der Woude F, Goulmy E. Tissue distribution of human minor histocompatibility antigens. Ubiquitous versus restricted tissue distribution indicated heterogenity among human cytotoxic T lymphocyte-defined non-MHC antigens. J Immunol. 1992; 149:1788–1794. 1380540

[12] Dickinson A, Wang X, Sviland L, Vyth-Dreese F, Jackson G, Schumacher T et al In situ dissection of the graft-versus-host activities of cytotoxic T cells specific for minor histocompatibility antigens. Nat Med. 2002; 8:410–414. 11927949 10.1038/nm0402-410

[13] Spierings E, Gras S, Reiser JB, Mommaas B, Almekinders M, Kester MG, et al Steric hindrance and fast dissociation explain the lack of immunogenicity of the minor histocompatibility HA-1Arg Null allele. J Immunol. 2009; 182:4809–4816. 10.4049/jimmunol.0803911 19342659

[14] Marijt W, Veenhof W, Goulmy E, Willemze R, van Rood J, Falkenburg J. Minor histocompatibility antigens HA-1, -2, and −4, and HY-specific cytotoxic T-cell clones inhibit human hematopoietic progenitor cell growth by a mechanism that is dependent on direct cell-cell contact. Blood. 1993; 82:3778–3785. 8260714

[15] van der Harst D, Goulmy E, Falkenburg J, Kooij-Winkelaar Y, van Luxemburg-Heijs S, Goselink H, et al Recognition of minor histocompatibility antigens on lymphocytic and myeloid leukemic cells by cytotoxic T-cell clones. Blood. 1994; 83:1060–1066. 8111046

[16] Falkenburg J, Goselink H, van der Harst D, van Luxemburg-Heijs S, Kooy-Winkelaar Y, Faber L, et al Growth inhibition of clonogeneic leukemic precursor cells by minor histocompatibility antigen-specific cytotoxic T lymphocytes. J Exp Med. 1991; 174:27–33. 2056279 10.1084/jem.174.1.27 PMC2118892

[17] Wilke M, Dolstra H, Maas F, Pool J, Brouwer R, Falkenburg J, et al Quantification of the HA-1 gene product at the RNA level; relevance for immunotherapy of hematological malignancies. Hematol J. 2003; 4:315–320. 14502255 10.1038/sj.thj.6200318

[18] Holloway PA, Kaldenhoven N, van Dijk M, Bloem AC, de Lau W, van der ZR, et al Susceptibility of malignant plasma cells to HA-1(H) specific lysis suggests a role for the minor histocompatibility antigen HA-1 in the graft-versus-myeloma effect. Leukemia. 2004; 18:1543–1545. 15322561 10.1038/sj.leu.2403445

[19] Hambach L, Nijmeijer B, Aghai Z, Schie M, Wauben M, Falkenburg J, et al Human cytotoxic T lymphocytes specific for a single minor histocompatibility antigen HA-1 are effective against human lymphoblastic leukaemia in NOD/scid mice. Leukemia. 2006; 20:371–374. 16357839 10.1038/sj.leu.2404056

[20] Marijt W, Heemskerk M, Kloosterboer F, Goulmy E, Kester M, van der Horn M, et al Hematopoiesis-restricted minor histocompatibilty antigens HA-1- or HA-2-specific T cells can induce complete remissions of relapsed leukemia. Proc Natl Acad Sci USA. 2003; 100:2742–2747. 12601144 10.1073/pnas.0530192100 PMC151411

[21] Mutis T, Brand R, Gallardo D, van Biezen A, Niederwieser D, Goulmy E. Graft-versus-host driven graft-versus-leukemia effect of minor histocompatibility antigen HA-1 in chronic myeloid leukemia patients. Leukemia. 2010; 24:1388–1392. 10.1038/leu.2010.115 20508613

[22] Spierings E, Kim YH, Hendriks M, Borst E, Sergeant R, Canossi A, et al Multicenter analyses demonstrate significant clinical effects of minor histocompatibility antigens on GvHD and GvL after HLA-matched related and unrelated hematopoietic stem cell transplantation. Biol Blood Marrow Transplant. 2013; 19:1244–1253. 10.1016/j.bbmt.2013.06.001 23756210

[23] Hobo W, Broen K, van der Velden WJ, Greupink-Draaisma A, Adisty N, Wouters Y, et al Association of disparities in known minor histocompatibility antigens with relapse-free survival and graft-versus-host disease after allogeneic stem cell transplantation. Biol Blood Marrow Transplant. 2013; 19:274–282. 10.1016/j.bbmt.2012.09.008 23022467 PMC3553241

[24] Spierings E, Drabbels J, Hendriks M, Pool J, Spruyt-Gerritse M, Claas F, et al A uniform genomic minor histocompatibility antigen typing methodology and database designed to facilitate clinical applications. PLoS ONE. 2006; 1:e42 17183671 10.1371/journal.pone.0000042 PMC1762400

[25] Mutis T, Gillespie G, Schrama E, Falkenburg J, Moss P, Goulmy E. Tetrameric HLA class I-minor histocompatibility antigen peptide complexes demonstrate minor histocompatibility antigen-specific cytotoxic T lymphocytes in patients with graft-versus-host disease. Nat Med. 1999; 5:839–842. 10395333 10.1038/10563

[26] Mutis T, Verdijk R, Schrama E, Esendam B, Brand A, Goulmy E. Feasibility of immunotherapy of relapsed leukemia with ex vivo-generated cytotoxic T lymphocytes specific for hematopoietic system-restricted minor histocompatibility antigens. Blood. 1999; 93:2336–2341. 10090944

[27] van Halteren AG, Jankowska-Gan E, Joosten A, Blokland E, Pool J, Brand A, et al Naturally acquired tolerance and sensitization to minor histocompatibility antigens in healthy family members. Blood. 2009 114:2263–72. 10.1182/blood-2009-01-200410 19506299 PMC3402366

[28] Goulmy E, Gratama JW, Blokland E, Zwaan FE, van Rood JJ. A minor transplantation antigen detected by MHC-restricted cytotoxic T lymphocytes during graft-versus-host disease. Nature. 1983; 302:159–161. 6186923 10.1038/302159a0

[29] Dupriez B, Morel P, Demory JL, Lai JL, Simon M, Plantier I, et al Prognostic factors in agnogenic myeloid metaplasia: a report on 195 cases with a new scoring system. Blood. 1996; 88:1013–1018. 8704209

[30] Glucksberg H, Storb R, Fefer A, Buckner C, Neiman P, Clift R, et al Clinical manifestations of graft-versus-host disease in human recepients of marrow from HL-A-matched sibling donors. Transplantation. 1974; 18:295–304. 4153799 10.1097/00007890-197410000-00001

[31] Shulman HM, Sullivan KM, Weiden PL, McDonald GB, Striker GE, Sale GE, et al Chronic graft-versus-host syndrome in man. A long-term clinicopathologic study of 20 Seattle patients. Am J Med. 1980; 69:204–217. 6996481 10.1016/0002-9343(80)90380-0

[32] Toes RE, van der Voort EI, Schoenberger SP, Drijfhout JW, van Bloois L, Storm G, et al Enhancement of tumor outgrowth through CTL tolerization after peptide vaccination is avoided by peptide presentation on dendritic cells. J Immunol. 1998; 160:4449–4456. 9574550

[33] Bijker MS, van den Eeden SJ, Franken KL, Melief CJ, van der Burg SH, Offringa R. Superior induction of anti-tumor CTL immunity by extended peptide vaccines involves prolonged, DC-focused antigen presentation. Eur J Immunol. 2008; 38:1033–1042. 10.1002/eji.200737995 18350546

[34] Stern M, Ruggeri L, Mancusi A, Bernardo ME, de Angelis C, Bucher C, et al Survival after T cell-depleted haploidentical stem cell transplantation is improved using the mother as donor. Blood. 2008; 112:2990–2995. 10.1182/blood-2008-01-135285 18492955 PMC2962448

[35] Verdijk R, Pool J, van der Keur M, Naipal A, van Halteren A, Brand A, et al Pregnancy induces minor histocompatibility antigen-specific cytotoxic T cells: implications for stem cell transplantation and immunotherapy. Blood. 2004; 103:1961–1963. 14592836 10.1182/blood-2003-05-1625

[36] Dierselhuis MP, Jankowska-Gan E, Blokland E, Pool J, Burlingham WJ, van Halteren AG, et al HY immune tolerance is common in women without male offspring. PLoS ONE. 2014; 9(3):e91274 10.1371/journal.pone.0091274 24646895 PMC3960116

[37] Spierings E, Vermeulen C, Vogt M, Doerner L, Falkenburg J, Mutis T, et al Identification of HLA class II-restricted H-Y specific T helper epitope evoking CD4+ T-helper cells in H-Y-mismatched transplantation. Lancet. 2003; 362:590–591. 12944060 10.1016/S0140-6736(03)14191-8

[38] Vogt M, van den Muijsenberg J, Goulmy E, Spierings E, Kluck P, Kester M, et al The DBY gene codes for an HLA-DQ5-restricted human male-specific minor histocompatibility antigen involved in graft-versus-host disease. Blood. 2002; 99:3027–3032. 11929796 10.1182/blood.v99.8.3027

[39] Boeck S, Hamann M, Pihusch V, Heller T, Diem H, Rolf B, et al Kinetics of dendritic cell chimerism and T cell chimerism in allogeneic hematopoietic stem cell recipients. Bone Marrow Transplant. 2006; 37:57–64. 16258529 10.1038/sj.bmt.1705217

[40] Andersen MH. The targeting of immunosuppressive mechanisms in hematological malignancies. Leukemia. 2014; 28:1784–1792. 10.1038/leu.2014.108 24691076

[41] Riches JC, Davies JK, McClanahan F, Fatah R, Iqbal S, Agrawal S, et al T cells from CLL patients exhibit features of T-cell exhaustion but retain capacity for cytokine production. Blood. 2013; 121:1612–1621. 10.1182/blood-2012-09-457531 23247726 PMC3587324

[42] Borchers S, Bremm M, Lehrnbecher T, Dammann E, Pabst B, Wolk B, et al Sequential anti-cytomegalovirus response monitoring may allow prediction of cytomegalovirus reactivation after allogeneic stem cell transplantation. PLoS ONE. 2012; 7(12):e50248 10.1371/journal.pone.0050248 23272059 PMC3521740

[43] Spierings E, Hendriks M, Absi L, Canossi A, Chhaya S, Crowley J, et al Phenotype frequencies of autosomal minor histocompatibility antigens display significant differences among populations. PLoS Genet. 2007; 3(6):e103 17604453 10.1371/journal.pgen.0030103 PMC1904367

[44] Wiedemann B, Klyuchnikov E, Kroger N, Zabelina T, Stahl T, Zeschke S, et al Chimerism studies with quantitative real-time PCR in stem cell recipients with acute myeloid leukemia. Exp Hematol. 2010; 38:1261–1271. 10.1016/j.exphem.2010.08.006 20851159

[45] Rufer N, Starobinski M, Chapuis B, Gratwohl A, Jeannet M, Helg C, et al Clinical consequences of sensitisation to minor histocompatibility antigens before allogeneic bone marrow transplantation. Bone Marrow Transplant. 1998; 22:895–898. 9827818 10.1038/sj.bmt.1701466

[46] Bonnet D, Warren E, Greenberg P, Dick J, Riddell S. CD8+ minor histocompatibility antigen-specific cytotoxic T lymphocyte clones eliminate human acute myeloid leukemia stem cells. Proc Natl Acad Sci USA. 2001; 96:8639–8644. 10.1073/pnas.96.15.8639 PMC17569 10411928

[47] Rosinski KV, Fujii N, Mito JK, Koo KK, Xuereb SM, Sala-Torra O, et al DDX3Y encodes a class I MHC-restricted H-Y antigen that is expressed in leukemic stem cells. Blood. 2008; 111:4817–4826. 10.1182/blood-2007-06-096313 18299450 PMC2343610

[48] Bonnet D, Dick JE. Human acute myeloid leukemia is organized as a hierarchy that originates from a primitive hematopoietic cell. Nat Med. 1997; 3:730–737. 9212098 10.1038/nm0797-730

[49] Matsui W, Wang Q, Barber JP, Brennan S, Smith BD, Borrello I, et al Clonogenic multiple myeloma progenitors, stem cell properties, and drug resistance. Cancer Res. 2008; 68:190–197. 10.1158/0008-5472.CAN-07-3096 18172311 PMC2603142

[50] Martinez-Climent JA, Fontan L, Gascoyne RD, Siebert R, Prosper F. Lymphoma stem cells: enough evidence to support their existence? Haematologica. 2010; 95:293–302. 10.3324/haematol.2009.013318 20139392 PMC2817033

