# Supplementary material for: Possible Role of Minor H Antigens in the Persistence of Donor Chimerism after Stem Cell Transplantation; Relevance for Sustained Leukemia Remission
Source: PLoS One. 2015 Mar 16;10(3):e0119595. doi: 10.1371/journal.pone.0119595 (PMC4361395; doi:10.1371/journal.pone.0119595)
Supplement: S1 Appendix — (DOCX) [file pone.0119595.s001.docx]

S1 APPENDIX

Overall, this study included 10 patients having received reduced-intensity (n=8) or myeloablative conditioning (n=2: patients 3,7) before transplantation of G-CSF-mobilized peripheral blood stem cells (PBSC, n=8) or bone marrow (BM, n=2: patients 7,8). Four patients were transplanted from HLA-matched sibling donors (MSD) and six patients were transplanted from HLA-matched unrelated donors (MUD). Three male patients (patients 3-5) received a sex-mismatched graft. GvHD prophylaxis consisted of cyclosporin A (CsA), methotrexate (MTX), mycophenolic acid (MMF), steroids and antibodies. Immune suppression was stopped in 8 patients (patient 1,2,4-6,8-10) during the observation period, 4 patients (patient 3,5-7) had episodes of acute GvHD, 3 patients (patient 4,5,10) had newly occurring chronic GvHD timely separated from acute GvHD and 5 patients (patients 5,6,9,10) received DLIs.

Patient 1

A 41 year old woman with AML (FAB M4/M5, complex karyotype) in second complete remission (CR2) received SCT from a female MUD after FLAMSA/TBI/Cy/ATG conditioning. GvHD prophylaxis consisted of CsA and MMF. Immune suppression ended on day 80 and blood collection ended on patient’s request on day 125. On day 142 she received a prophylactic DLI (5x10^5^ CD3+ cells/kg). Meningeal relapse was diagnosed on day 149 which was treated with chemotherapy, radiation and additional DLIs (1x10^6^ CD3+ cells/kg on day 257, 5x10^6^ CD3+ cells/kg on day 296 and 5x10^6^ CD3+ cells/kg on day 380). The patient died on day 391.

Patient 2

A 58 year old woman with PMF received SCT from her sister after Fludarabin/Busulphan/ATG conditioning. GvHD prophylaxis consisted of CsA and MTX. The post-transplant period was complicated by a veno-occlusive disease responding to Prociclide. Sample analysis ended on day 670. The patient is alive and well.

Patient 3

A 18 year old man with ALL (pro-B ALL, normal karyotype) in CR1 received SCT from his sister after TBI/etoposide/thymoglobuline conditioning. GvHD prophylaxis consisted of CsA and steroids. The post-transplant period was complicated by GvHD IV^o^ with slight skin and severe gut involvement starting on day 29. Regression of symptoms occurred after high dose steroids (500mg/d), Basiliximab and a course of anti-lymphocyte globulin on day 43. Steroids were increased and infliximab was started on day 90 resulting in a temporary reduction of symptoms. On day 126 ileus symptoms started together with candida sepsis. On day 149 the bowel was depressurized by an ileostoma. On day 183 onset of psychotic symptoms associated with cerebral lesions in the magnetic resonance tomography. Regressive GvHD symptoms allowed tapering of immune suppression. Sample analysis ended on day 203. On day 248, stereotactic brain biopsy revealed cerebral fungus infection. The patient died on day 275.

Patient 4

A 39 year old man with AML (FAB M5, t(9;11)) in first partial remission (PR1) received SCT from his sister after FLAMSA/TBI/Cy/ATG conditioning. GvHD prophylaxis consisted of CsA and MMF. The post-transplant period was uncomplicated. Chronic GvHD I-II^o^ of skin and mouth was diagnosed on day 120 which disappeared without specific treatment. Sample analysis ended on day 958. The patient is alive and well.

Patient 5

A 59 year old man with AML (FAB M0, complex karyotype) in PR1 received SCT from a female MUD after FLAMSA/TBI/Cy/ATG conditioning. GvHD prophylaxis consisted of CsA and MMF. The post-transplant period was complicated by veno-occlusive disease responding to Prociclide. GvHD II^o^ (skin and gut) was diagnosed on day 12 which responded to steroids. The post-transplant period was uncomplicated until on day 453 bone marrow relapse was diagnosed. The patient received chemotherapy and 3 DLIs (1x10^7^ CD3+ cells/kg on day 496, 2x10^7^ CD3+ cells/kg on day 528, 1x10^8^ CD3+ cells/kg on day 552). Chronic GvHD of skin, mouth and liver was diagnosed on day 579 and the patient was treated with CsA and steroids. Sample analysis ended on day 598. The patient died on day 654 due to chronic lung GvHD and multi organ failure.

Patient 6

A 51 year old man with sAML/MDS (FAB M6, complex karyotype) in CR2 received SCT from a male MUD after FLAMSA/TBI/Cy/ATG conditioning. GvHD prophylaxis consisted of CsA and MMF. The post-transplant period was complicated by a skin GvHD I^o^ starting on day 45. On day 503, bone marrow relapse was diagnosed and treated with chemotherapy. The patient received a 1st DLI (1.4x10^7^ CD3+ cells/kg) on day 554 and a 2nd DLI (5x10^7^ CD3+ cells/kg) on day 582. A new chemotherapy was started on day 619 leading to aplasia. On day 665 a second SCT from another donor was performed. Sample analysis ended on day 629. The patient died on day 984 after second relapse.

Patient 7

A 48 year old woman with CLL (complex karyotype) with PR2 received BMT from a male MUD after TBI/Fludarabin/Cyclophosphamide/Campath conditioning. GvHD prophylaxis consisted of CsA. The post-transplant period was complicated by a herpes encephalitis (onset on day 25), bacteraemias and venous thrombosis. GvHD II^o^ (skin) started on day 67 which responded to steroids but reoccurred on day 149 after tapering of immune suppression. Fungal pneumonia was diagnosed on day 241. Chronic skin GvHD increased on day 267. Sample analysis ended on day 265. The patient died on day 290 due to sepsis.

**Patient 8**

A 37 year old woman with severe aplastic anemia with no response to ATG/CsA upon relapse after initially successful ATG/CsA treatment received 71 months after first diagnosis BMT from her sister after TBI/Fludarabin/Cyclophosphamide conditioning. GvHD prophylaxis consisted of CsA. The post-transplant period was uncomplicated. Sample analysis ended on day 686. The patient is alive and well.

Patient 9

A 41 year old man with CLL (normal karyotype, non-mutated VH-Gen) in PR2 received SCT from his brother after Fludarabin/Cyclophosphamide/ATG conditioning. GvHD prophylaxis consisted of CsA and MTX. The post-transplant period was uncomplicated. Due to persistently low donor chimerism, the patient received several DLIs (1st DLI 1x10^6^ CD3+ cells/kg on day 237, 2nd DLI 5.8x10^6^ CD3+ cells/kg on day 266, 3rd DLI 1.1x10^7^ CD3+ cells/kg on day 306) without clinical response. On day 349, the patient presented with LN swellings. The patient received 13 courses Rituximab from day 364 to 530 resulting in an increase of donor chimerism. Sample analysis ended on day 453. Additional DLIs (4th DLI 3.1x10^7^ CD3+ cells/kg on day 593, 5th DLI 5.8x10^7^ CD3+ cells/kg on day 644, 6th DLI 7.9x10^7^ CD3+ cells/kg on day 868) were applied without clinical response. The patient is currently treated with Rituximab.

Patient 10

A 46 year old man with PMF received SCT from a male MUD after Fludarabin/Busulphan/ATG conditioning. GvHD prophylaxis consisted of CsA and MTX. The post-transplant period was uncomplicated. Due to persistently low donor chimerism, the patient received several DLIs (1st DLI 1x10^6^ CD3+ cells/kg on day 834, 2nd DLI 5x10^6^ CD3+ cells/kg on day 862, 3rd DLI 1x10^7^ CD3+ cells/kg on day 889). Due to signs of chronic GvHD (skin, liver, lung) on day 958, the patient received steroids until day 1381. The patient converted to complete donor chimerism. Sample analysis ended on day 1162. The patient is alive and well.
