# Supplementary figures and images for: Possible Role of Minor H Antigens in the Persistence of Donor Chimerism after Stem Cell Transplantation; Relevance for Sustained Leukemia Remission
Source: PLoS One. 2015 Mar 16;10(3):e0119595. doi: 10.1371/journal.pone.0119595 (PMC4361395; doi:10.1371/journal.pone.0119595)

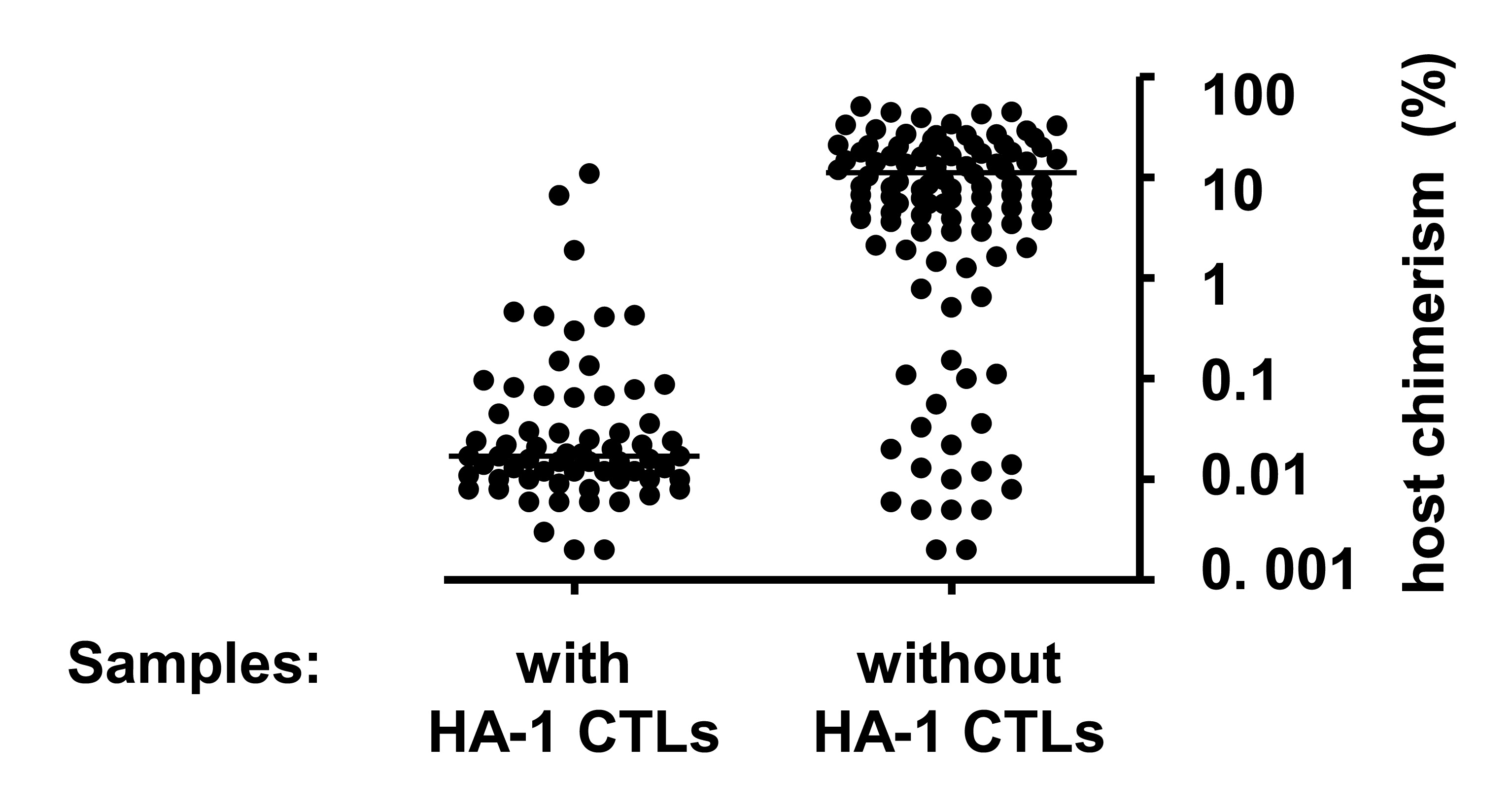

Supplement: S1 Fig — The horizontal lines indicate the median values. (TIF) [file pone.0119595.s002.tif]

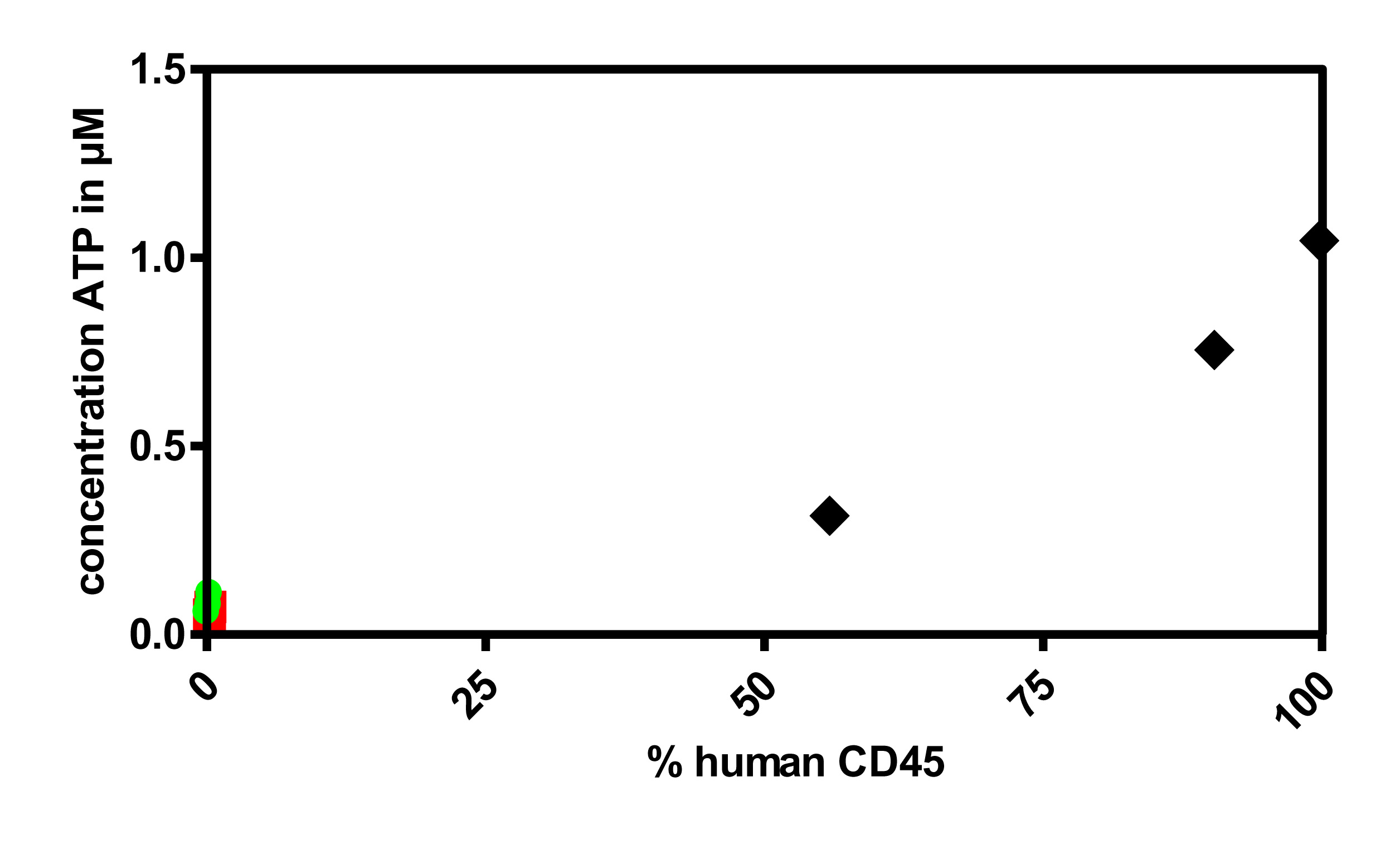

Supplement: S2 Fig — BM of NOD/SCID mice transplanted with UCB derived CD34+ cells preincubated with CMV (black diamonds), HA-1 (red boxes) or alloA2 CTLs (green circles) was subjected 16 weeks after transplantation to an HALO progenitor cell assay and flowcytometry for human CD45. This assay determines intracellular ATP levels as measure for cell proliferation in response human cytokine stimuli. ATP is detected after 7 days of in vitro culture with bioluminscence in relative light units (RLU). Exact ATP concentrations were calculated based on the RLU in relation to a standard ATP curve. The %human CD45+ cells after 7 days of in vitro culture was determined for every HALO sample. X-axis: ATP concentration in μM; Y-axis: % human CD45% cells. (TIF) [file pone.0119595.s003.tif]

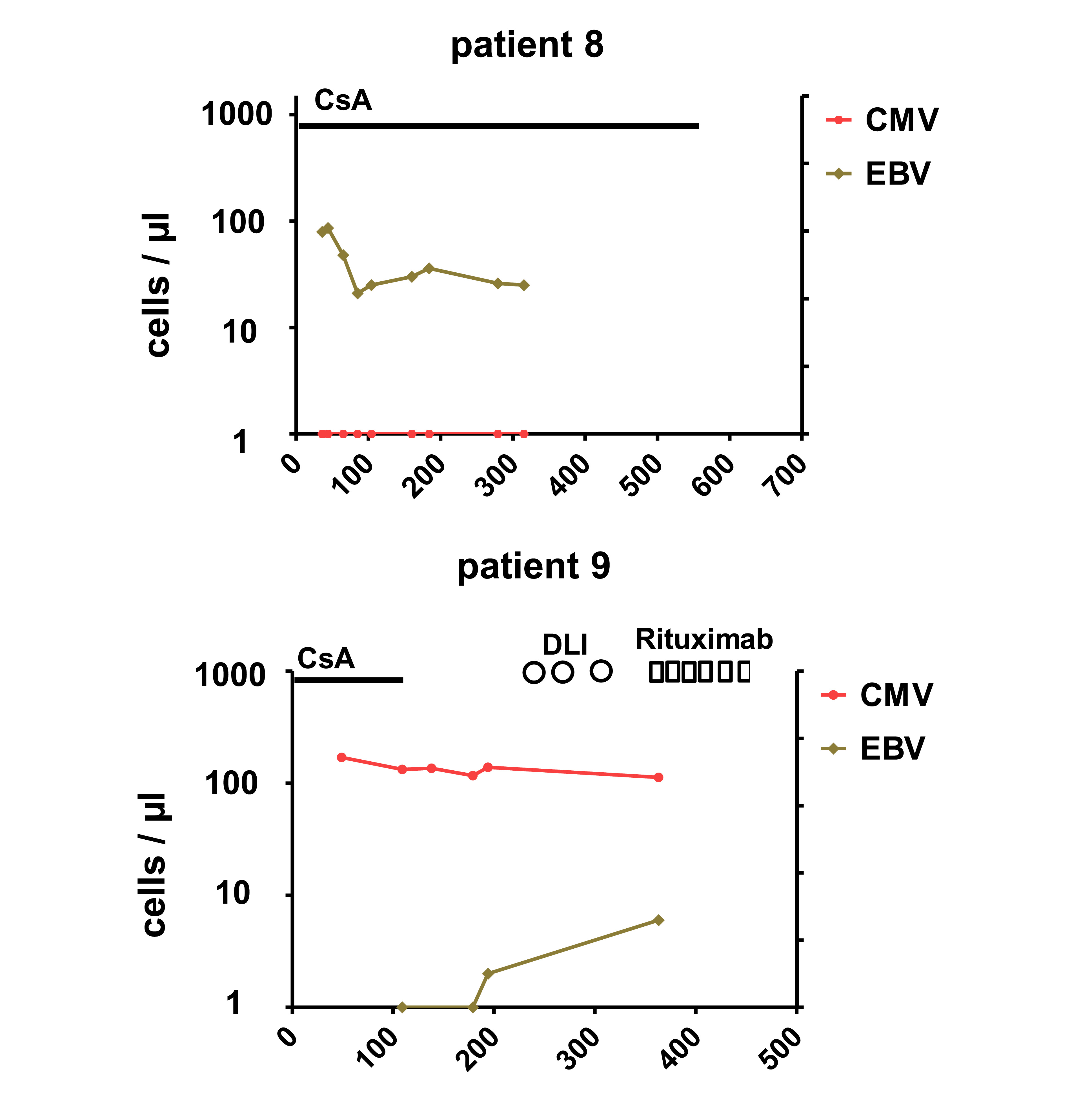

Supplement: S3 Fig — HLA-A2-restricted CMV (red line) and EBV CTLs (grey line) were detected by tetramer staining of fresh blood as described in [42]. Thus, data cannot be directly compared with the data performed on frozen samples presented in Fig. 1. (TIF) [file pone.0119595.s004.tif]
